# Chloroform in Cholera

**Published:** 1849-02

**Authors:** 


*Chloroform in Cholera.*—In Edinburgh it appears that chloroform failed to *cure* cholera; in other hands it has been more successful. The following memoranda of cases treated in the Peckham House Asylum, show favorahle results:

| | | |
|---|---|---|
| Total number of | | |
| Malignant cases | 42 | |
| Relapses | 6 | |
| | | 48 |
| Recoveries | 33 | |
| Deaths | 15 | |
| Treated by chloroform, as the sheet-anchor: | | |
| Cases | 37 | |
| Relapses | 6 | |
| | | 43 |
| Recoveries | 31 | |
| Deaths | 12 | |

Two of these fatal cases were dying before chloroform was resorted to.

The mortality throughout England and Scotland, has been about two-thirds.—*Western Lancet.*

